# PET Imaging of Carotid Atherosclerosis: Methodology, Implications, and Applications in Neurovascular Disease

**DOI:** 10.1161/STROKEAHA.125.050399

**Published:** 2026-01

**Authors:** Shiv Bhakta, John J McCabe, Jason M Tarkin, Mohammed M Chowdhury, Jessica Redgrave, James HF Rudd, Peter J Kelly, Elizabeth A Warburton, Nicholas R Evans

**Affiliations:** 1 Department of Clinical Neurosciences, University of Cambridge, Cambridge, UK; 2 School of Medicine, University College Dublin, Dublin, Ireland; 3 Stroke Clinical Trials Network Ireland, Catherine McAuley Centre, Nelson Street, Dublin, Ireland; 4 Division of Cardiorespiratory Medicine, Department of Medicine, University of Cambridge, Cambridge, UK; 5 Department of Vascular Surgery, University of Cambridge, Cambridge, UK; 6 Department of Clinical Neurology, Sheffield Teaching Hospitals NHS Trust, Sheffield, UK

## Abstract

Carotid atherosclerosis is a significant cause of incident and recurrent ischemic stroke, with risk not solely related to the degree of luminal stenosis. Multimodal imaging approaches, including PET/CT and PET/MRI, can provide anatomical and molecular evaluation of the atherosclerotic plaque *in vivo*. Plaque pathophysiology – including the key processes of inflammation and microcalcification – may help characterize stroke risk beyond conventional anatomical assessment alone.

This review discusses the use of PET in the investigation of carotid atherosclerosis, including methodological considerations, its contributions to our understanding of the underlying disease processes, and how imaging can be used in interventional trials. The clinical implications and potential future applications of PET in the assessment and treatment of cerebrovascular disease are also examined.

## Graphical abstract

**Graphic abstract:**
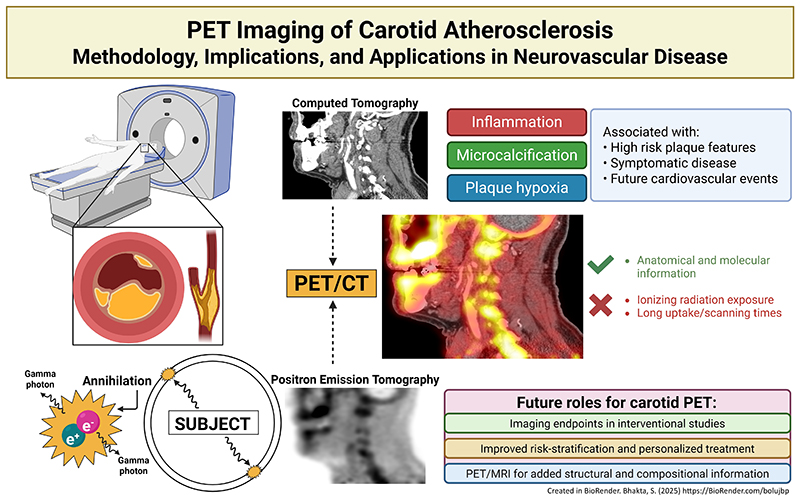

## Non-standard Abbreviations and Acronyms

AS: active segment
cSUV: corrected standardized uptake value
cSUV_max_: corrected maximum standardized uptake value
CT: computed tomography
CTA: computed tomography angiography
DOTATATE: [1,4,7,10-tetraazacyclododecane-N,N’,N”,N”’-tetraacetic acid]-D-Phe^1^,Tyr^3^-octreotate
FDG: fluorodeoxyglucose
FMISO: fluoromisonidazole
HR: hazard ratio
MRI: magnetic resonance imaging
NaF: sodium fluoride
PET: positron emission tomography
PVE: partial volume effect
ROI: region of interest
SCAIL: symptomatic carotid atheroma inflammation lumen-stenosis
SNR: signal-to-noise ratio
SSTR2: somatostatin receptor type 2 SUV standardized uptake value
SUV_max_: maximum standardized uptake value
SUV_mean_: mean standardized uptake value
TBR: target-to-background ratio
TBR_max_: maximum target-to-background ratio
TBR_mean_: mean target-to-background ratio
TSPO: translocator protein

## Introduction

Ischemic stroke is a significant cause of morbidity, mortality, and healthcare costs worldwide^1^, with increasing numbers of strokes between 1970 and the present^1^. Up to 37% of all ischemic strokes are related to large artery atherosclerosis^2,3^, which is also the stroke etiology most associated with early recurrence^4^. At the population level, the prevalence of moderate to severe carotid disease increases with age^5^, and hence represents an important etiology for the global ageing population.

Atherosclerosis is a systemic vascular disease involving the development of lipid-rich atheroma (plaques)^6^. Although the degree of luminal stenosis represents an important feature relating to subsequent stroke risk and selection for surgical intervention^7^, advances in imaging techniques in recent years have facilitated more detailed assessment of high-risk carotid plaque morphology and pathophysiology. Amongst these techniques is positron emission tomography (PET), a nuclear medicine modality that enables pathophysiology – including those related to atherogenesis – to be detected and quantified non-invasively *in vivo*.

In this review, we discuss methodological considerations when performing PET in the carotid arteries, how this technique has aided our understanding of the underlying pathophysiology, and emerging and future uses of PET for risk-stratification and evaluation of therapeutic interventions for atherosclerosis.

### Principles of PET in the carotid artery

#### General principles of PET

PET uses radioisotope-labelled molecular ligands (tracers) to detect and quantify specific metabolic processes of interest^8^. β^+^ decay of the radionuclide produces positrons that interact with electrons, resulting in an annihilation reaction that produces γ photons that are detected by scintillation detectors. PET imaging is sensitive to picomolar concentrations of the tracer used^9^, enabling evaluation of changes in pathophysiology over time, including responses to therapeutic interventions. This is further facilitated by high inter-rater agreement in tracer quantification^10^.

In addition to these general considerations, different radiotracers require different uptake times to generate images of appropriate quality^11^. Certain tracers also have additional requirements to ensure image interpretability, such as a period of fasting and ensuring euglycemia when imaging with fluorodeoxyglucose (FDG)^11^, which may reduce its tolerability from a patient perspective (Table 1).

14

**Figure 1:**
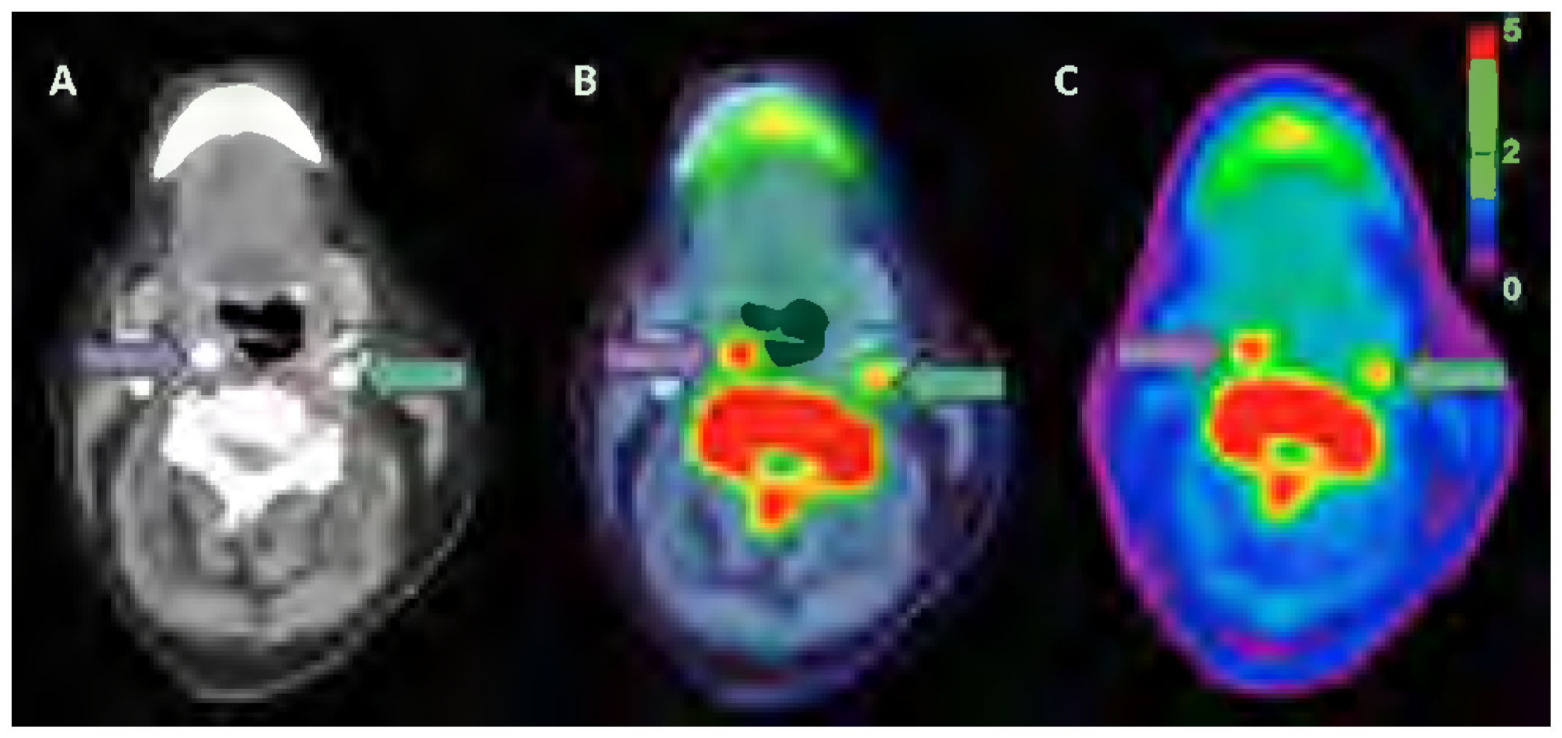
Tracer uptake in symptomatic disease. **A**: axial CTA, **B**: axial PET/CTA, **C**: axial PET showing a symptomatic right carotid artery (purple arrow) and an asymptomatic left carotid artery (green arrow). Adapted with permission from Evans et al, 2020^14^.

There is an inherent limit to the spatial resolution of PET, due to the random motion of positrons produced by the radionuclide prior to their interaction with electrons^12^. Different radionuclides have different positron energies, and consequently different positron ranges, with lower energies giving improved spatial resolution^12^. The lack of anatomical data from PET means co-registration with alternative modalities (such as computed tomography, CT, or magnetic resonance imaging, MRI) is required to provide anatomical localization^13^ (Figure 1, showing CT data in panel A, PET data in panel C, and the co-registered PET-CT image in panel B^14^), and to allow attenuation correction of the raw gamma scintigraphy, as different tissues cause differing attenuation of the incident γ photons^15^.

#### Methodological considerations specific to the carotid arteries

Specific to the carotid arteries, analysis of endarterectomy specimens offers *ex vivo* histological correlation and validation of *in vivo* carotid PET imaging^8^. Compared to other arterial territories, and given the spatial and temporal resolution of PET, the carotid arteries provide a superficial and relatively immobile target for imaging. The size of carotid plaques is advantageous in relation to partial volume effects (PVE), the ability to determine accurately tracer uptake in small-volume structures^16^ that is related to the spatial resolution of PET^16^, a particular issue in atherosclerosis imaging when dealing with relatively small plaque volumes. Assessment of plaque tracer uptake can also be affected by spillover from adjacent structures that also accumulate the tracer (such as the thyroid gland)^17^, as well as blood-pooling of the tracer^16^. Renal impairment may also affect plaque uptake through reducing excretion of the circulating tracer^18^, and methods for accounting for this are discussed below.

Carotid PET is usually combined with CT for attenuation correction, and CT angiography (CTA) for anatomical localization. CT is commonly used in the clinical setting, allowing comparison with previous or subsequent imaging, but is associated with additional radiation exposure^15^. The use of iodinated contrast for angiography may pose a risk of adverse reactions, including in those with significant renal impairment. Using MRI for attenuation correction may avoid additional radiation exposure, and provide additional information to identify high-risk plaque features, such as intraplaque haemorrhage^19^, lipid-rich necrotic core^19^, or a thin/ruptured fibrous cap^20^. PET/MRI is increasingly used as a research tool in atherosclerosis, though its availability remains limited and has several potential technical issues specific to hybrid imaging with MRI^20^.

The timing of PET after symptoms is important, as the intensity of the underlying process may change with time and/or treatment^10^. Although FDG is the most widely used radiotracer in carotid imaging, its non-specific uptake has necessitated optimization of tracer doses and uptake times to ensure adequate radiotracer uptake in the plaque^21^, whilst minimizing artifacts related to spillover and PVE. More specific tracers for inflammation, as well as tracers targeting other pathophysiological processes of interest have elucidated mechanisms of plaque disruption, and are discussed further below.

#### Evaluating tracer uptake

Several semi-quantitative methods exist to measure tracer uptake. Standardized uptake values (SUV) are calculated as the activity in a region of interest (ROI), normalized for body weight and injected radiotracer dose. The maximum (SUV_max_) or mean (SUV_mean_) uptake within the ROI have both been used, but have issues when considering uptake in atherosclerotic plaques. SUV_max_ may be less accurate and reproducible due to PVE, spillover, and inhomogeneous plaque uptake^22^, while SUV_mean_ can be difficult to calculate accurately due to difficulties in defining the edge of the plaque^22^.

**Figure 2:**
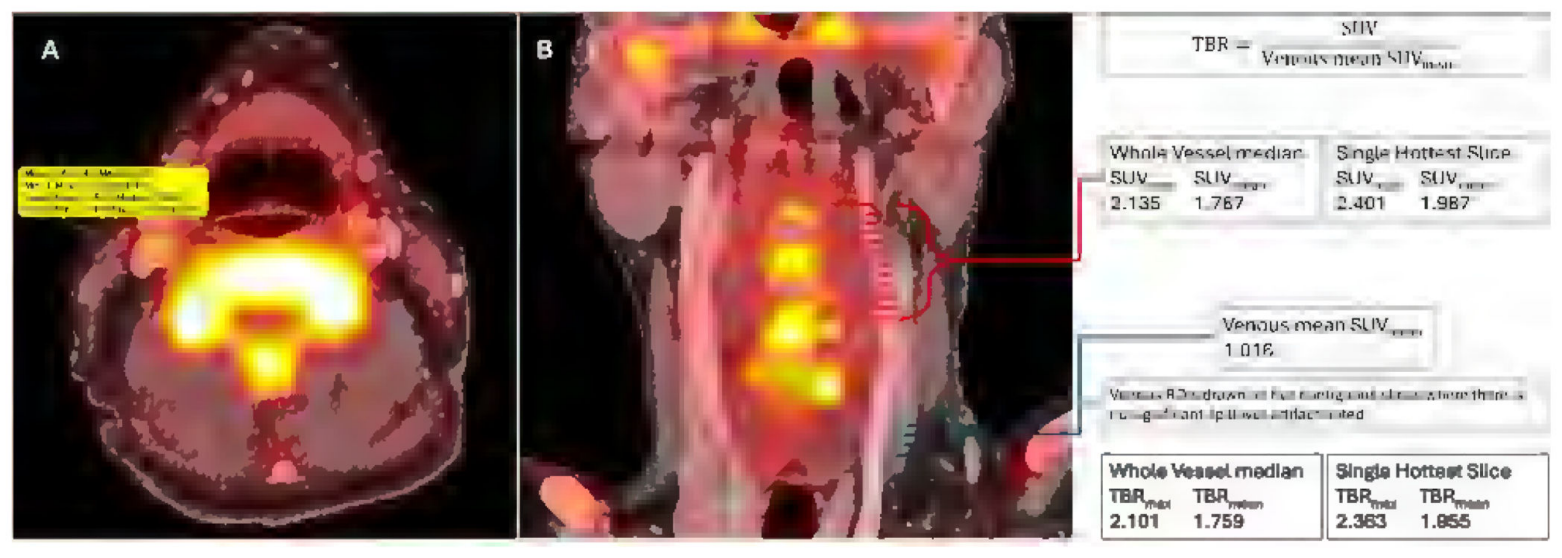
Evaluating tracer uptake. **A:** axial PET/CTA with an example ROI, showing SUV data. **B:** coronal PET/CTA with arterial ROIs displayed around the carotid bifurcation (red lines), and ROIs in a proximal vein unaffected by spillover from adjacent structures (blue lines). TBR statistics are calculated by taking the relevant arterial SUV statistic and dividing by the mean of the SUV_mean_ obtained from the venous ROIs.

For vascular PET imaging, the need to correct for blood-pool tracer activity has led to the development of alternative methods to quantify plaque uptake. The target-to-background ratio (TBR) is calculated as the ratio of the ROI SUV to the SUV_mean_ of venous blood^17^ (Figure 2). TBRs are reproducible and potentially less sensitive to changes in imaging parameters such as tracer uptake time^11^. Another alternative is the corrected SUV (cSUV)^23^, where cSUV_max_ is the ROI SUV_max_ minus venous SUV_mean_. Fewer studies report cSUV compared to TBR, and more data is required comparing cSUV and TBR to assess their relative reproducibility and sensitivity^23^.

**Figure 3:**
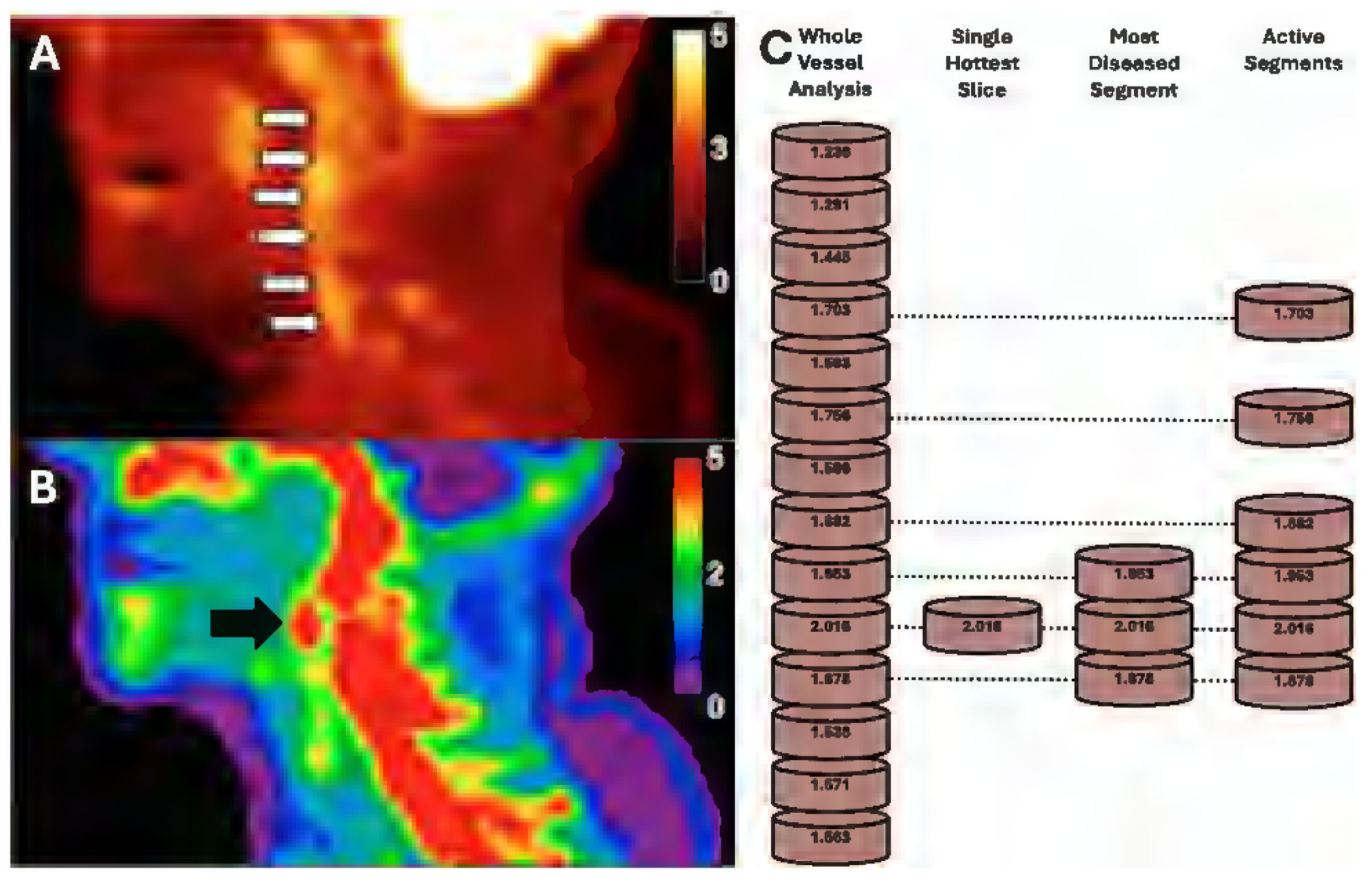
Common approaches to measure arterial tracer uptake. **A:** sagittal FDG-PET showing diffuse uptake in the symptomatic carotid (white arrows), **B:** sagittal NaF-PET showing focal uptake in the symptomatic carotid (black arrow), **C:** diagram demonstrating methods to quantify arterial uptake. Whole Vessel analysis uses the average ROI uptake value, and the Single Hottest Slice uses the maximum value of the segments being evaluated. The Most Diseased Segment uses the mean ROI uptake of the Single Hottest Slice and its adjacent two ROIs, while the Active Segment method takes all ROI uptake values above a prespecified limit – in this case ≥1.6. Panels A and B adapted with permission from Evans et al, 2020^14^.

Choosing the appropriate measurement for the process being imaged is also important. In diffuse processes, analysis of a whole vessel or segment may be appropriate, such as the average TBR_max_ of the whole vessel. This may be more robust to spillover artifact and image noise, but less able to detect focal changes^11^. In processes expected to cause highly focal changes, it may be more useful to compare areas of high uptake, such as the single hottest slice (the highest uptake value), the most diseased segment (the mean uptake of the single hottest slice and its immediately adjacent ROIs) or assessment of active segments (AS, ROIs with an uptake value greater than a prespecified value)^11^ (Figure 3). These approaches are more sensitive in detecting focal changes, at the risk of increased susceptibility to artifact. When using the AS approach, developing a meaningful threshold value may be difficult in terms of clinical relevance and imaging sensitivity. These approaches tend to use ROIs from axial PET slices, with the slice thickness used varying between studies. The spatial resolution of PET limits the utility of decreasing the slice thickness below 2-3mm.

A position statement on FDG-PET in atherosclerosis provides recommendations regarding protocols and quantification of tracer uptake, allowing comparison between studies^11^. However, this consensus is limited to FDG and further standardized approaches to other tracers would be advantageous.

### How PET has facilitated the identification of carotid pathophysiology i*n vivo*

#### Inflammation

FDG is a glucose analogue, resistant to glycolysis and retained in cells upon phosphorylation^9^. It is therefore a non-specific marker of metabolic activity, used clinically to identify increased glucose metabolism, such as with cancer or inflammation. Incidentally noted arterial uptake led to the investigation of FDG-PET for detecting and characterizing inflammation in atherosclerosis^24,25^. FDG uptake varies between plaques^26^, and is associated with macrophage-rich areas on autoradiography^26^ and histology^27^ of excised surgical specimens. FDG uptake is additionally strongly associated with morphological characteristics associated with a high risk of plaque rupture on histology^28^ and imaging^29^, including irregular plaque surfaces^28^, low-attenuation plaque^28^ and lipid-rich necrotic cores^29^. High-risk plaque features have been associated with higher FDG uptake even in plaques with <50% stenosis^30^, highlighting the limitations of an entirely stenosis-based assessment of atherosclerotic plaques. Reflecting these findings, a meta-analysis of carotid FDG studies demonstrated increased uptake in symptomatic plaques compared to asymptomatic plaques^31^.

Increased carotid FDG uptake is associated with vascular risk factors^32–34^, inflammatory biomarkers^35,36^, and higher rates of recent^37^ and historic^38^ cardiovascular events. FDG uptake is also associated with plaque progression as measured by increasing mean vessel wall thickness^39^. FDG-PET has also elucidated the therapeutic mechanisms underlying treatment effects: statins reduce plaque FDG uptake, independent of change in low density lipoprotein levels^40^, with a dose-response effect^41^.

The importance of plaque macrophage burden, and the non-specific nature of FDG uptake, has led to interest in tracers with higher specificity. These include translocator protein (TSPO) ligands, such as ^11^C-PK11195, and somatostatin receptor type 2 (SSTR2) ligands, such as DOTATATE ([1,4,7,10-tetraazacyclododecane-N,N’,N”,N”’-tetraacetic acid]-D-Phe^1^,Tyr^3^-octreotate). With PK11195, symptomatic plaques showed higher TBR^42^, and colocalization with macrophages in endarterectomy specimens^43^. DOTATATE-PET has been used primarily in imaging coronary atherosclerosis, but has shown promise in the carotid arteries, where uptake was higher in symptomatic plaques compared to the contralateral artery^44^. Uptake has been found to correlate strongly with macrophage-rich areas on histology^44^, and with SSTR2^44^ and CD163 expression^45^. *Ex-vivo* DOTATATE binding is associated with SSTR2 expressing cells and vulnerable plaque features^46^. Further carotid studies validating the utility of DOTATATE for evaluating macrophage burden may lead to DOTATATE supplanting FDG due to reduced requirements for fasting or euglycemia, an improved signal-to-noise ratio (SNR), and ^68^Ga not requiring a cyclotron for production.

Of note, non-atherosclerotic diseases can lead to vascular inflammation and stroke, including giant cell arteritis, where FDG-PET has a diagnostic role. Vessel wall uptake in giant cell arteritis tends to be more diffuse and symmetrical when compared with uptake in atherosclerosis^47^.

#### Other metabolic processes

Microcalcification within atherosclerotic plaques occurs through the actions of osteoblast-like cells, derived from vascular smooth muscle cells in response to inflammatory cytokines^48^. Microcalcification can provoke further inflammation^49^, and plaque rupture through increasing mechanical stress at the fibrous cap^50^. Microcalcification (typically <50μm) is below the spatial resolution of CT or MRI *in vivo*, but may be visualized using sodium fluoride (NaF) PET. As with inflammation, vessel wall microcalcification was noted incidentally, with microcalcification not correlated with macrocalcification visible on CT^51^, or in endarterectomy specimens^52^, suggesting NaF accumulates at areas of active microcalcification, rather than at sites of large calcium deposits.

NaF uptake is associated with vascular risk factors^53^, high-risk plaque features on histology and *in vivo* imaging^54^, and with symptomatic disease^55^. In dual NaF-FDG studies, arterial lesions rarely demonstrated both FDG and NaF uptake at the same site^56^, and noncalcified lesions showed no correlation between FDG and NaF uptake, while concordant uptake was noted in mildly (r=0.7) and severely calcified (r=0.4) lesions^57^. Symptomatic plaques also showed higher NaF uptake compared to asymptomatic plaques^58^, but no correlation with FDG uptake, in contrast to previous studies, suggesting inflammation and microcalcification are related, but distinct processes, and these techniques can identify different stages of plaque evolution^59^.

^18^F-fluoromisonidazole (FMISO) is a tracer which accumulates after selective reduction within hypoxic cells^60^. Hypoxia occurs in atherosclerotic plaques due to increased oxygen demand from inflammatory cells, with an increasing degree of hypoxia associated with increasing necrotic core size and plaque thickness^61^. FMISO and FDG uptake are correlated, with uptake higher in symptomatic compared to asymptomatic plaques^62^.

#### Predicting cerebrovascular events with PET

Pathophysiology observed using carotid PET may indicate the future risk of neurovascular events. In individuals with cancer undergoing FDG-PET, increased vascular FDG uptake was associated with a higher risk of future events^63^. After adjustment for cardiovascular risk factors and degree of luminal stenosis, both inflammation^64,65^ and microcalcification^66^ are strongly associated with higher rates of stroke recurrence, with a hazard ratio (HR) of 2.19 per unit increase in SUV_max_ with FDG^65^, and an odds ratio of 1.24 per unit increase in TBR_max_ with NaF^66^. FDG signal is also associated with microembolic signals, suggesting one mechanism for higher recurrence rates^67^.

The symptomatic carotid atheroma inflammation lumen-stenosis (SCAIL) score, incorporating plaque FDG SUV_max_ (SUV_max_ <2, 0 points; 2–2.99, 1 point; 3–3.99, 2 points; ≥4, 3 points) with luminal stenosis (<50%, 0 points; 50%–69%, 1 point; ≥70%, 2 points), improved prediction of early stroke recurrence compared to stenosis severity alone, with a HR of 2.40 per 1-point increase^68^, stroke recurrence at 5 years, with an HR of 2.73 per 1-point increase^69^, and in a population with uncertain benefit from revascularization^70^. Another group found FDG SUV_max_ alone was a better predictor of 90-day recurrent events, compared to SCAIL scores^71^. Addition of FDG uptake data to Framingham risk scores improved incident cardiovascular disease prediction in individuals undergoing cancer surveillance^72^. Together, these results demonstrate stenosis severity alone is insufficient for accurate risk discrimination, that PET may help identify those who would most benefit from revascularization. This may include those with multiple potentially co-existing stroke etiologies, and where there is equipoise between revascularization and optimal medical therapy, including in those with asymptomatic stenosis.

### Emerging and future applications

#### Using imaging endpoints in interventional studies

Definitive randomized controlled trials of novel pharmacotherapies for atherosclerosis require large sample sizes, significant time, and financial investment. PET has potential value in translational research, using surrogate imaging endpoints to demonstrate the potential efficacy of therapeutic agents at targeting the processes visualized, over shorter timescales^11^. Given its high sensitivity and inter-reader reliability, PET may facilitate early-phase trials by enabling small sample sizes and shorter follow-up^73^, with therapies failing to demonstrate reductions in the target processes potentially signifying futility of further assessment.

PET is well-suited for use in clinical trials, as inflammation and microcalcification are associated with recurrent stroke, a clinically relevant endpoint. However, the relationship between PET signal change and consequent changes to clinical outcomes is unknown. If a drug is to demonstrate improved clinical outcomes, a large change in uptake on PET would likely be required^17^. PET-based imaging studies of these interventions would also not supplant trials for safety outcomes, although this requirement would likely be reduced in drug-repurposing studies, where the safety of the drug in question would be previously established.

#### PET-based endpoints in carotid atherosclerosis

Imaging endpoints can be used to confirm the mechanism or target of action of a therapeutic agent, or to compare relative effects of different agents. A meta-analysis of the role of statins in reducing vascular inflammation showed significant reductions in FDG uptake with high-intensity statins, which was not seen with low- or moderate-intensity statin therapy^74^. The roles of other lipid-modification therapies on vascular inflammation has been assessed, with alirocumab treatment causing a reduction in carotid TBR_max_^75^, while treatment with dalcetrapib did not reduce FDG uptake compared to placebo^76^. The use of anti-inflammatory agents have shown variable results, with inhibitors of p38 mitogen-activated protein kinase^77^ and 5-lipoxygenase^78^ not demonstrating significant changes in FDG uptake compared to placebo. In non-FDG studies, liraglutide did not demonstrate any significant changes in signal compared to placebo in a carotid DOTATATE-PET study^79^ (although benefits have been noted in coronary studies^80^), while rosuvastatin was associated with significant reductions in plaque NaF uptake^81^, although this study did not include a comparator group.

PET can compare the effects of different interventions on plaque biology. High-intensity statins had greater anti-inflammatory effects compared with lower-intensity therapy^41^, as did pioglitazone compared to glimepiride in a statin-naïve population^82^. Ongoing trials are using PET in the investigation of new and repurposed therapies, including comparing the anti-inflammatory effects of alirocumab and ezetimibe^83^, and colchicine^84^. Wider use of established and novel tracers, and further understanding of the *in vivo* plaque processes, such as the role of elevated lipoprotein (a), may lead to more targeted therapies being considered, and assessed using PET, as a pathway into routine clinical use.

#### Technical considerations with PET/MRI

Assessment of carotid atherosclerosis with MRI may be preferable to CT, due to avoiding excess radiation exposure^15^ and intravenous contrast agents for those with adverse reactions or renal impairment^20^. Vascular wall imaging using MRI may enable further characterization of plaque morphological features associated with an increased risk of rupture or recurrent disease^20^. However, vascular wall imaging generally requires higher magnetic field-strength (three tesla or greater^85^), which has issues with cost^20^, availability^20^, sensitivity to motion artifact^86^, and compatibility issues in those with metallic implants^20^. Non-contrast MRI angiography sequences tend to require longer imaging times than contrast-enhanced equivalents, while contrast-enhanced sequences have potential issues related to gadolinium accumulation, especially with serial imaging^87^. The longer imaging times and narrower bore size with MRI compared to CT lead to issues with patient tolerability^20^.

MRI, unlike CT, does not provide direct information regarding photon attenuation^88^, requiring alternative methods to generate attenuation maps^88,89^. Rigid and stationary radiofrequency coils can have predefined attenuation maps generated, but for flexible or mobile coils – which vary in position or shape – specific corrections must be performed to ensure accurate tracer uptake readings^90^. Different coils can have effects on both MRI and PET signal, and optimization is required to improve image quality^91^. This is especially the case in carotid plaque imaging, where specific coils may be required for adequate resolution at the level of the vessel wall. Such coils may not be routinely available in clinical settings and are associated with significant expense. PET equipment must be adapted to withstand the magnetic fields, and these fields can also affect the trajectory of positrons, leading to imaging artifact if corrections are not applied^20^.

Separate MRI and PET-CT can cause difficulties in anatomical colocalization, while the use of hybrid PET-MRI scanners solves this issue. When comparing radiotracer uptake between PET-MRI and PET-CT systems, there may be an underestimation of PET parameters with MRI systems due to the differing image reconstruction methods used, and longer imaging times with MRI affecting tracer clearance^92,93^. Given these issues, it is unclear if direct comparisons between imaging outcomes can be made^93^. As PET-MRI technology and experience matures, more precise anatomical and physiological identification of high-risk plaque features may be possible. This may provide direct correlation between pharmacological interventions and their effects on both plaque pathophysiology and high-risk morphological features.

#### Potential future applications of carotid PET

As highlighted above, PET has potential roles both in risk-stratification and assessing efficacy of novel medical interventions. Validation of novel tracers may further our understanding of the pathophysiology underpinning plaque vulnerability, and their transition to stability^94^. PET-based endpoints for early-phase studies may improve research efficiency, with rejection of candidate medications that demonstrate limited change with serial PET, while those with significant effects can move more rapidly to phase III studies. PET may also be used to demonstrate trafficking of radiolabeled drug to the plaque, providing imaging evidence of targeting *in vivo*. Comparing data obtained on PET-CT and PET-MRI systems is also important, given potential differences in radiotracer uptake, to ensure appropriate comparisons can be performed^93^. Radiomic analysis, with PET along with other modalities, may provide additional benefit in understanding plaque pathophysiology, including through identifying factors associated with plaque rupture from *ex vivo* imaging of endarterectomy specimens, or correlating PET activity with *in vivo* CT/MRI imaging, allowing associations with metabolic activity to be elucidated.

Wider use of PET in clinical practice – likely resulting in increased throughput of imaging and reduced scanning costs – may benefit individualized risk prediction in those with carotid disease by identifying those at high risk of recurrence not meeting the current criteria for surgical intervention, such as those with low-grade stenosis where other high-risk plaque features are noted (with potential implications for identifying such etiology in individuals with embolic stroke of undetermined source. Furthermore, it may have a role in identifying those with significant carotid stenosis who have high surgical risk but no high-risk plaque features beyond the degree of stenosis, and who may benefit more from optimizing medical management^95,96^.

#### Limitations of carotid PET imaging

Routine clinical use of PET is limited by three factors: technical factors, issues integrating PET into workflows, and standardizing outputs. Plaque imaging may be susceptible to artifact due to spillover and PVE, and may also be affected by attenuation artifact (and artifact in the co-registered image) due to metallic implants. Movement between acquisition of attenuation maps and PET data may decrease accuracy of attenuation correction and anatomical co-registration, hindering interpretation.

Increasing clinical use of PET in atherosclerosis requires strict scheduling and logistical support to ensure simultaneous availability of the patient, radiotracer and scanner. The ability to generate radiotracers locally and on demand would facilitate this but requires dedicated facilities and expertise. Tracer uptake time and pre-scan preparations increase healthcare contact time, and reduce patient tolerability, and the associated ionizing radiation exposure and contrast administration for angiography may preclude imaging in certain patients.

Standardization of protocols for imaging acquisition and interpretation is required, to ensure appropriate and accurate reporting of relevant data, and to facilitate comparison between studies and more robust meta-analysis. Current interpretation methods are time-intensive, reducing reporting throughput. Together, these factors affect the cost-effectiveness, and therefore clinical application, of PET in the assessment of atherosclerosis.

## Conclusions

PET imaging of carotid plaques can accurately characterize pathophysiological processes *in vivo*, and may be harnessed to develop our understanding of plaque physiology and evaluate the impact of novel therapeutic agents in modifying plaque characteristics. For clinical practice, carotid PET has potential future applications in improving recognition of symptomatic plaques, and personalized risk-stratification in stroke.

## Figures and Tables

**Table 1:** Technical considerations with selected radiotracers used in carotid PET imaging

Tracer	Uptake time (minutes)	Advantages	Disadvantages	Standard dose	Effective dose (PET only)
^18^F-FDG	120 (recommendation) 60-190 (previous studies)	High sensitivity Plaque uptake highly correlated with macrophage burden Good tracer availability and experience with use Plaque uptake indicates culprit plaques and relates to future recurrence risk High reproducibility between studies Changes in uptake with intervention can be detected by 3-4 months	6 hours fasting pre-scan and blood glucose ≤7.2mmol/L required for optimal imaging May require withholding metformin prior to scan Potential poor image quality in those with diabetes mellitus Potential spillover artifact from thyroid gland (or muscle/lymph nodes when measuring venous SUV), increased blood-pooling with renal insufficiency Low uptake specificity Increased carotid uptake compared to other arterial territories	3-4MBq/kg (recommendation) 185-925MBq (previous studies)	0.019mSv/MBq
^18^F-NaF	60-90 (recommendation) 60-180 (previous studies)	High specific binding and sensitivity Rapid target uptake and clearance from plasma Plaque uptake indicates culprit plaques and relates to future recurrence risk Short uptake times	Potential spillover artifact from adjacent bony structures Lower tracer availability and clinical experience than with FDG	185MBq (recommendation) 125-370MBq (previous studies)	0.017mSv/MBq
^18^F-FMISO	120-180	Plaque uptake indicates symptomatic plaques	Less data regarding its use in vascular PET Low SNR Potential spillover artifact from head/neck musculature	300MBq	0.014mSv/MBq
^68^Ga- DOTATATE ^64^Cu- DOTATATE	60 (^68^Ga) 60-300 (^64^Cu)	High specific binding High SNR (improved with shorter uptake times) Plaque uptake indicates culprit plaques ^68^Ga: tracer production does not require a cyclotron	Potential spillover artifact from thyroid and salivary glands ^68^Ga: higher positron range	150MBq (^68^Ga) 150-200MBq (^64^Cu)	0.029mSv/MBq (^68^Ga) 0.03mSv/MBq (^64^Cu)
